# Different Hemodynamic Characteristics and Resulting in Different Risks of Rupture Between Wide-Neck and Narrow-Neck Aneurysms

**DOI:** 10.3389/fneur.2022.868652

**Published:** 2022-04-25

**Authors:** Heng Wei, Qi Tian, Kun Yao, Jianfeng Wang, Peibang He, Yujia Guo, Wenrui Han, Wenhong Gao, Mingchang Li

**Affiliations:** ^1^Department of Neurosurgery, Renmin Hospital of Wuhan University, Wuhan, China; ^2^Department of Neurosurgery, Jingzhou Central Hospital, Jingzhou, China

**Keywords:** fluid dynamics, hemodynamic, wide-neck aneurysms, narrow-neck aneurysms, subarachnoid hemorrhage

## Abstract

**Objective:**

This study aimed to determine the ruptured rate and hemodynamic difference between wide-neck aneurysms (WNAs) and narrow-neck aneurysms (NNAs), as well as the hemodynamic parameters of risk factors for aneurysm rupture.

**Methods:**

A total of 121 cases of intracranial aneurysms (IAs) were studied retrospectively between January 2019 and April 2021 at Renmin Hospital of Wuhan University. Intracranial aneurysms were classified into four types: ruptured wide-neck aneurysms (RWNAs), unruptured wide-neck aneurysms (UWNAs), ruptured narrow-neck aneurysms (RNNAs), and unruptured narrow-neck aneurysms (UNNAs). The Chi-square test was used to compare differences in rupture ratios. The clinical characteristics and hemodynamics were analyzed statistically to reveal the rupture risk factors. Moreover, significant parameters were subjected to binary logistic regression analysis to identify the independent predictive factors. The receiver operating characteristic (ROC) curve was performed to obtain cutoff values.

**Results:**

WNAs ruptured more frequently than NNAs (*P* = 0.033). Ruptured intracranial aneurysms (RIAs) were characterized by significantly higher intra-aneurysmal pressure (IAP), wall shear stress (WSS), wall shear stress gradient (WSSG), and lower normalized wall shear stress (NWSS) than unruptured intracranial aneurysms (UIAs). RWNAs had higher IAP, WSS, and lower NWSS than UWNAs (*P* < 0.05). RNNAs had higher IAP, Streamwise WSSG and lower NWSS compared to UNNAs (*P* < 0.05). Binary logistic regression revealed that IAP and WSS were independent predictive risk factors for WNAs rupture, with cut-off values of 405.5 and 6.66 Pa, respectively. Also, IAP was an independent predictive risk factor for NNA rupture, with a cut-off value of 255.3 Pa.

**Conclusions:**

Wide-neck aneurysms and narrow-neck aneurysms have diverse hemodynamics, which prompts a higher rupture ratio for WNAs. IAP could characterize the rupture risk in both WNAs and NNAs independently, but WSS could only predict the rupture risk in WNAs. This research might assist neurosurgeons with fostering a more sensible strategy for the treatment of IAs.

## Introduction

Intracranial aneurysms (IAs) are vascular pathologies characterized by dilatation of the arterial wall in the brain. Previous research had observed IAs in ~2–5% of the adult population (1), and IAs at risk of rupture may result in a distinct disease known as subarachnoid hemorrhage (SAH), which is associated with high mortality and morbidity (2). These pieces of evidence imply that treating UIAs before they rupture could extremely be beneficial (3). Observational natural history studies of UIAs, on the other hand, have revealed that only a small percentage of all UIAs patients rupture (4), with a total rupture risk of 1.2% within 5 years of diagnosis (5). In this view, an individualized assessment of the rupture risk is essential for balancing the risks of operative treatment and observation.

Several studies have looked at risk factors for the rupture of IAs, including size, shape, location, age, hypertension, diabetes, smoking, and family history (6–8). These elements, however, have an extremely restricted capacity to predict whether IAs will rupture (9). Much consideration has been paid in recent years to the relationship between hemodynamics (10), vessel wall enhancement (11), radiomics (12), and aneurysm formation or rupture. Image-based computational fluid dynamics (CFD) modeling can help find both the hemodynamic value and the correlation of the value with ruptured IAs (13). The hemodynamics parameters may play an important role in the mechanisms underlying the rupture of cerebral aneurysms (14), though their precise role is still unknown and the results contradict each other sometimes (15). Therefore, a more accurate model to predict the rupture risk of IAs is warranted.

Clinical evidence shows the following as the most common preoperative classification of IAs by neurosurgeons: ruptured or unruptured, wide-necked or narrow-necked. Endovascular therapeutic strategies for ruptured and unruptured IAs have been recommended (16). Endovascular therapy for WNAs frequently necessitates stent-assisted coiling therapy (17), which has been linked to an increased risk of stroke due to stent thrombosis (18) and postoperative bleeding (19). However, we discovered that previous hemodynamic studies on aneurysms only looked at the risk of rupture and did not differentiate between WNAs and NNAs. Therefore, the purpose of this study was to compare the hemodynamic differences between WNAs and NNAs to assist neurosurgeons in developing more appropriate surgical strategies for IAs.

## Materials and Methods

### Patient Selection

We retrospectively examined the medical records and imaging data of 101 patients who were diagnosed with IAs and were admitted to our hospital between January 2019 and April 2021. Cerebral Digital subtraction angiography (DSA) was used to diagnose all aneurysms. We obtained complete clinical and serial imaging data of all patients. Two neurosurgeons with over 10 years of experience in the interpretation of cerebral angiograms and the endovascular treatment of IAs confirmed the findings.

Inclusion criteria: (1) aneurysms with a diameter ranging from 3 to 10 mm; (2) complete clinical, radiological, and hemodynamic data; (3) sac intracranial aneurysm. Exclusion criteria: (1) patients with malignant tumors, malignant hypertension, and severe systemic disease; (2) pseudoaneurysm, inflammatory aneurysms, traumatic aneurysms, dissecting aneurysms, aneurysms with arteriovenous malformations, multiple aneurysms, and fusiform aneurysms; (3) 3D-DSA images with poor quality for CFD.

Aneurysms with a diameter of 3–10 mm were selected to reduce statistical deviation in hemodynamic results. In clinics, IAs in this diameter range were the most common (20, 21). Research evidence also shows that aneurysm size is closely related to rupture risk, and IAs with a significantly large size have a high rupture risk (22). Microaneurysms were excluded from the study to reduce the deviation owing to their unique morphology, rupture risk, and prognosis (23, 24). Aneurysms with diameters <3 and >10 mm were, therefore, excluded from this study.

### Patient Groups

The 3D model of the aneurysm and parent vessels could be tumbled freely and measured on our workplace system. Two-dimensional (2D) angiography was performed after selecting the best angle to expose aneurysm neck and aneurysm sac in 3D model. Morphological variables of aneurysms were measured by 2D-DSA imaging, including diameter (D, the maximum distance from the center of aneurysm neck to a point on the sac) and neck width (N, the maximum diameter in the neck plane). The morphological parameters of the aneurysms were measured and independently recorded by two neuroradiologists with more than 10 years of experience.

The literature has great variability regarding the definition of a WNA, D/N used for the definition ranged from 1 to <2 (1, 1.2, 1.5, 1.6, 1.8, and <2). The most prevalent definition is a neck diameter of ≥4 mm or a D/N of <2 and our study adopts this standard, while NNAs had a D/N of ≥2 (25).

Patients in each group were further divided into ruptured or unruptured aneurysms based on the presence of SAH. SAH was diagnosed using brain CT. Lumbar puncture was considered mandatory to confirm the diagnosis in cases where SAH was suspected clinically but brain CT was negative (26). Two neurosurgeons confirmed the findings.

### Image Model

Standard transfemoral artery catheterization was used for all catheter angiographies. All 3D images were obtained through DSA using a Siemens Integris biplane device (Siemens Healthineers, Forchhein,Germany), whereas rotational angiograms were performed 2 s after 5-s contrast injection, with a 18 ml contrast agent and a 360° rotation. The corresponding images were reconstructed into 3D modeling on the workstation and then exported in DICOM format. The aneurysm dome, neck, and related parent arteries were measured using conventional angiographic images and reference markers included in the view.

### Aneurysm Modeling

DICOM format image data imported into the software Mimics medical 21.0 (Materialise, Leuven, Belgium) to segment geometries and obtain the preliminary 3D models, and then import the software 3-matic medical 13.0 (Materialise, Leuven, Belgium) in STL format for model repair and smoothing. The software ANSYS Fluent 2021 R2 and CFD-post 2021 R2 (ANSYS Inc., USA) was used for simulations of hemodynamics and for generating the solution. Model mesh division was carried out in Fluent, controlling the maximum size of poly-hexcore mesh 0.1 mm. In order to accurately obtain the hemodynamic parameters of the model surface (inner vessel wall), six boundary layers were specially set during mesh generation, and the thickness of the first layer was 0.01 mm, the layer-by-layer growth rate is 1.2. Each geometry was meshed to create 0.5–1.5 million volume elements for subsequent fluid dynamics computation. The types of boundary conditions were velocity inlet and pressure outlet, respectively. The pulsating velocity waveform measured by transcranial Doppler ultrasound was used as the inflow boundary condition. The flow waveform was scaled to obtain the mean inlet velocity of 0.5 m/s under pulsating conditions. The outlet was set at zero pressure and flow rate through each outlet artery was proportional to the cube of its diameter. The vessel was modeled as a rigid wall, and the blood flow was modeled as an incompressible Newtonian fluid with constant temperature and laminar flow, resulting in an Navier–Stokes equation approximate blood flow. A density of 1,060 kg/m^3^ and a dynamic viscosity of 0.0035 Pa·s were specified for each simulation. Monitor check convergence absolute criteria was set ≤ 1 × 10^−4^. Finally, the calculation time was set to last for three cardiac cycles, with the result of the third cycle reaching a stable state after 200-time steps. Three pulsatile cycles were simulated to ensure that numerical stability had been reached, and the last cycle was taken as output. All data presented were time-averages over the third pulsatile cycle of flow simulation.

### Hemodynamic Parameter Calculation

The neck of the aneurysm was sketched on the reconstructed model. Several points in the neck were selected interactively and connected along a line with the shortest geodesic distance. The aneurysm opening was then triangulated, and the computational grid was divided into two regions, one for the aneurysm and one for the parent artery. Several blood flow variables defined by the wall, volume, or inlet of the aneurysm were quantified and used to characterize the hemodynamics of the aneurysm.

The following hemodynamic parameters were calculated: combined hemodynamic parameters (CHPs), normalized pressure (NP), normalized wall shear stress (NWSS), oscillatory shear index (OSI), IAP, relative residence time (RRT), streamwise wall shear stress gradient (WSSG), WSS, and WSSG. The average value was calculated for all hemodynamic parameters. WSS is the tangential pulling force acting on the vascular wall (13); IAP is the force energy with which blood strikes the inner wall of the aneurysm sac; NWSS is the ratio of aneurysm wall shear force to mean value of the parent artery wall shear force (13); WSSG is the amplitude of variation along the wall shear force direction (27); streamwise WSSG denotes flow acceleration and deceleration in the branches, creating positive and negative streamwise gradients in WSS (28); OSI is the drastic change in the direction of wall shear force during a cardiac cycle (13); RRT represents the time it takes for blood to stagnate around the vascular wall (22); NP represents the ratio of time-averaged pressure normalized by the parent vessel pressure (27); CHP is the weighted average of WSS and OSI (29). All hemodynamic parameters were computed using the methods described previously.

Many previous literatures have used micro-pressure probes to measure the pressure in the sac of aneurysms, while we measure the impact pressure of the blood flow on the inner wall of the aneurysm. The governing equation is as follows:


$$\begin{array}{lll} \;\nabla \cdot u & = & 0\\ \frac{\partial u}{\partial t}+u\cdot \nabla u & = & -\frac{1}{\rho }\nabla p+\frac{\mu }{\rho }\nabla^{2}u \end{array}$$

p stands for IAP in the manuscript, u is the velocity, ρ is the blood density. The blood parameters were defined by constant density ρ = 1,060 kg·m^−3^ and constant dynamic viscosity μ = 0.0035 Pa·s.

### Statistical Analysis

All statistical analyses were performed using two-tailed tests of SPSS Statistics 25 (IBM, Armonk, New York, USA). Continuous variables were analyzed using the independent *t*-test or Mann–Whitney *U* test and are presented as means ± SD or medians with interquartile ranges. Chi-square tests were used to analyze categorical variables, which are expressed as numbers. Binary logistic regression models were used to test the effect of different hemodynamic parameters on rupture status in univariate analysis, with a backward elimination procedure used to identify independent risk factors, and ROC analysis performed on independent risk factors to obtain cut-off values. A two-tailed *p* < 0.05 (95% confidence) denoted statistical significance.

## Results

### Different Rupture Risk Between WNAs and NNAs

The present study included 121 consecutive patient-specific aneurysms, including 53 RWNAs, 23 RNNAs, 22 UWNAs, and 23 UNNAs. We revealed through chi-square test that the ruptured ratio of WNAs was significantly higher than that of NNAs (*P* = 0.033).

### Clinical Characteristics of RIAs and UIAs

The RIAs comprised 26 males and 50 females, with a mean age of 59.42 ± 8.58 years. The UIAs were 16 males and 29 females, with a mean age of 58.22 ± 7.08 years. The results shows the clinical characteristics of RIAs and UIAs. There were no significant differences in age, sex, hypertension, coronary heart diseases, diabetes, atherosclerosis, alcohol consumption, or smoking (Table 1).

**Table 1:** Clinical characteristics of RIAs and UIAs.

**Variable**	**Ruptured**	**Unruptured**	***P*-value**
Age (years)	59.42 ± 8.58	58.22 ± 7.08	0.431
Gender
Male	26	16	0.997
Female	50	29	
Hypertension
Yes	46	21	0.291
No	30	24	
Diabetes
Yes	13	8	0.930
No	63	37	
CHD
Yes	7	1	0.094
No	69	44	
Atherosclerosis
Yes	6	3	0.479
No	70	42	
Drink
Yes	8	9	0.226
No	67	36	
Smoking
Yes	11	9	0.558
No	64	36	

*CHD, coronary heart disease*.

The clinical characteristics of RWNAs and UWNAs, RNNAs, and UNNAs were also compared. The results showed no significant differences between the groups in terms of age, sex, incidences of hypertension, coronary heart disease, diabetes, atherosclerosis, alcohol consumption, and smoking (Table 2).

**Table 2:** Clinical characteristics of RWNAs and UWNAs, RNNAs and UNNAs.

**Variable**	**RWNAs**	**UWNAs**	***P*-value**	**RNNAs**	**UNNAs**	***P*-value**
Age	60.25 ± 9.17	59.55 ± 7.68	0.754	57.52 ± 6.85	56.96 ± 6.36	0.773
(years)						
Gender						
Male	17	8	0.845	9	8	0.766
Female	36	14		14	15	
Hypertension						
Yes	36	10	0.070	10	14	0.562
No	17	12		13	9	
Diabetes						
Yes	9	6	0.317	4	2	0.227
No	44	16		19	21	
CHD						
Yes	6	1	0.275	1	0	0.323
No	47	21		22	23	
Atherosclerosis						
Yes	5	2	0.779	2	1	0.561
No	48	20		21	22	
Drink						
Yes	5	3	0.597	3	6	0.486
No	48	19		20	17	
Smoking						
Yes	8	4	0.744	3	5	0.718
No	45	18		20	18	

*RWNAs, ruptured wide-neck aneurysms; UWNAs, unruptured wide-neck aneurysms; RNNAs, ruptured narrow-neck aneurysms; UNNAs, unruptured narrow-neck aneurysms; CHD, coronary heart disease*.

### Hemodynamic Risk Factors Between RIAs and UIAs

Compared to UIAs, the RIAs group had significantly higher IAP (461.801 ± 150.023 vs. 264.146 ± 106.776 Pa, *P* < 0.001), WSS (6.495 ± 2.076 vs. 5.096 ± 1.054 Pa, *P* < 0.001), WSSG (730.568 ± 124.377 vs. 672.654 ± 95.541, *P* = 0.008), and lower NWSS (0.648 ± 0.157 vs. 0.765 ± 0.190, *P* < 0.001). Other hemodynamic parameters did not differ significantly between RIAs and UIAs (Table 3).

**Table 3:** Result of hemodynamic parameters between RIAs and UIAs.

**Variable**	**RIAs**	**UIAs**	***P***
CHP	0.118 ± 0.026	0.124 ± 0.023	0.181
NP	1.255 ± 0.157	1.275 ± 0.221	0.566
NWSS	0.648 ± 0.157	0.765 ± 0.190	<0.001
OSI	0.014 ± 0.015	0.013 ± 0.004	0.565
IAP(Pa)	461.801 ± 150.023	264.146 ± 106.776	<0.001
RRT	0.060 ± 0.018	0.054 ± 0.014	0.067
Streamwise WSSG	133.013 ± 40.069	124.099 ± 42.046	0.248
WSS (Pa)	6.495 ± 2.076	5.096 ± 1.054	<0.001
WSSG	730.568 ± 124.377	672.654 ± 95.541	0.008

*RIAs, rupture intracranial aneurysms; UIAs, unrupture intracranial aneurysms; CHP, combined hemodynamic parameters; NP, normalized pressure; NWSS, normalized wall shear stress; OSI, oscillatory shear index; IAP, intra-aneurysmal pressure; RRT, relative residence time; Streamwise WSSG, streamwise wall shear stress gradient; WSS, wall shear stress; WSSG, wall shear stress gradient*.

### Hemodynamic Parameters in UNNAs, RNNAs, UWNAs, and RWNA

Compared to UWNAs, the RWNAs had significantly higher IAP (521.718 ± 122.056 vs. 324.375 ± 111.379 Pa, *P* < 0.001), WSS (7.239 ± 1.504 vs. 5.485 ± 0.976 Pa, *P* < 0.001), and lower NWSS (0.641 ± 0.156 vs. 0.750 ± 0.189 *P* = 0.041). Other hemodynamic parameters did not differ significantly between UWNAs and RWNAs (Table 4).

**Table 4:** Results of hemodynamic parameters between RWNAs and UWNAs.

**Variable**	**RWNAs**	**UWNAs**	***P*-value**
CHP	0.116 ± 0.022	0.122 ± 0.027	0.330
NP	1.224 ± 0.143	1.215 ± 0.209	0.835
NWSS	0.641 ± 0.156	0.750 ± 0.189	0.041
OSI	0.015 ± 0.018	0.012 ± 0.004	0.395
IAP (Pa)	521.718 ± 122.056	324.375 ± 111.379	<0.001
RRT	0.057 ± 0.012	0.051 ± 0.011	0.067
Streamwise WSSG	131.331 ± 36.850	134.429 ± 55.814	0.778
WSS (Pa)	7.239 ± 1.504	5.485 ± 0.976	<0.001
WSSG	747.123 ± 128.225	706.720 ± 96.981	0.189

*RWNAs, ruptured wide-neck aneurysms; UWNAs, unruptured wide-neck aneurysms; CHP, combined hemodyn -amic parameters; NP, normalized pressure; NWSS, normalized wall shear stress; OSI, oscillatory shear index; IAP, intra-aneurysmal pressure; RRT, relative residence time; Streamwise WSSG, streamwise wall shear stress gradient; WSS, wall shear stress; WSSG, wall shear stress gradient*.

Compared to UNNAs, RNNAs had significantly higher IAP (323.723 ± 113.724 vs. 206.536 ± 62.158 Pa, *P* < 0.001), Streamwise WSSG (136.890 ± 47.342 vs. 114.218 ± 18.798, *P* = 0.038), and lower NWSS (0.664 ± 0.160 vs. 0.779 ± 0.193, *P* = 0.033). Other hemodynamic parameters did not differ significantly between RNNAs and UNNAs (Table 5).

**Table 5:** Results of hemodynamic parameters between RNNAs and UNNAs.

**Variable**	**RNNAs**	**UNNAs**	***P*-value**
CHP	0.123 ± 0.032	0.127 ± 0.018	0.643
NP	1.327 ± 0.167	1.332 ± 0.221	0.928
NWSS	0.664 ± 0.160	0.779 ± 0.193	0.033
OSI	0.013 ± 0.004	0.014 ± 0.002	0.101
IAP (Pa)	323.723 ± 113.742	206.536 ± 62.158	<0.001
RRT	0.068 ± 0.025	0.057 ± 0.017	0.121
Streamwise WSSG	136.890 ± 47.342	114.218 ± 18.798	0.038
WSS (Pa)	4.783 ± 2.223	4.725 ± 1.008	0.909
WSSG	692.418 ± 108.137	640.070 ± 83.714	0.073

*RNNAs, ruptured narrow-neck aneurysms; UNNAs, unruptured narrow-neck aneurysms; CHP, combined hemodynamic parameters; NP, normalized pressure; NWSS, normalized wall shear stress; OSI, oscillatory shear index; IAP, intra-aneurysmal pressure; RRT, relative residence time; Streamwise WSSG, streamwise wall shear stress gradient; WSS, wall shear stress; WSSG, wall shear stress gradient*.

### Binary Logistic Regression for WNAs and NNAs

The hemodynamic parameters related to NNAs and WNAs rupture were subjected to binary logistic regression analysis. IAP (OR = 1.013, 95% CI, 1.006–1.021, *p* < 0.001) and WSS (OR = 2.087, 95% CI, 1.202–3.594, *p* = 0.009) were found to be the independent predictive risk factors for IA rupture in the WNAs group. However, in the NNAs group, IAP (OR = 1.022, 95% CI, 1.006–1.039, *p* = 0.008) was the only independent predictive risk factor for IA rupture (Table 6).

**Table 6:** The results of binary logistic regression analysis for WNAs and NNAs.

**Group**	**Variable**	**OR (95%, CI)**	***P*-value**
	NWSS	0.190 (0.001, 6.856)	0.273
RWNAs vs. UWNAs	IAP	1.013 (1.006, 1.021)	<0.001
	WSS	2.087 (1.202, 3.594)	0.009
	NWSS	0.005 (0.000, 1.051)	0.052
RNNAs vs. UNNAs	IAP	1.022 (1.006, 1.039)	0.008
	Streamwise WSSG	1.023 (0.995, 1.053)	0.110

*RWNAs, ruptured wide-neck aneurysms; UWNAs, unruptured wide-neck aneurysms; RNNAs, ruptured narrow-neck aneurysms; UNNAs, unruptured narrow-neck aneurysms; NWSS, normalized wall shear stress; IAP, intra-aneurysmal pressure; WSS, wall shear stress; Streamwise WSSG, streamwise wall shear stress gradient*.

### The ROC Curve of Independent Risk Factors for WNAs and NNAs Rupture

**Figure 1:**
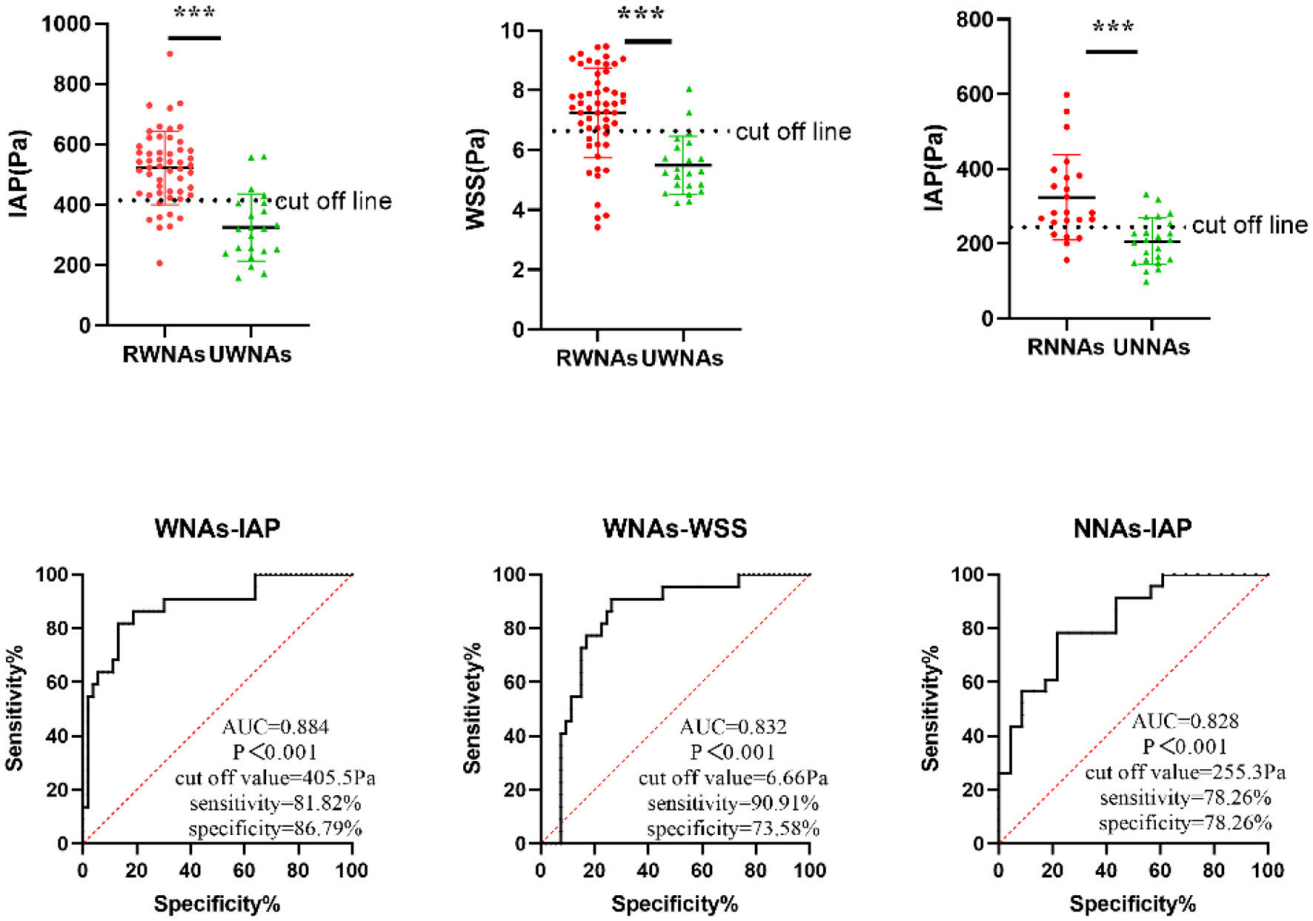
ROC curve of independent predictive hemodynamic parameters for NNAs and WNAs rupture. *** means *P* < 0.001.

The results of ROC analysis were showed that IAP (AUC = 0.884, *p* < 0.001) and WSS (AUC = 0.832, *p* < 0.001) had a diagnostic value for WNAs ruptured risk. the cut off values of IAP and WSS 405.5 and 6.66 Pa, respectively in WNAs group. IAP (AUC = 0.828, *p* < 0.001) had a diagnostic value for NNAs ruptured risk also. The cutoff values of IAP was 255.3 Pa in NNAs group (Figure 1).

## Discussion

Subarachnoid hemorrhage caused by ruptured aneurysms can have serious consequences in terms of morbidity and mortality (2). Meanwhile, surgical treatment of aneurysms, whether clipping or endovascular treatment, has a high rate of complications that result in death or disability (30). Therefore, understanding how to accurately evaluate the rupture risk and balance the risk between operation and observation is critical for UIAs. The most common factors considered by neurosurgeons in developing surgical strategies were the rupture risk and whether the IAs was wide neck. Of note, WNAs had a significantly higher surgical risk than NNAs. Previous studies on the risk of aneurysm rupture did not differentiate between WNAs and NNAs (8, 31, 32). In this study, we discovered that the rupture ratio of WNAs was significantly higher than that of NNAs by analyzing the continuous data from aneurysms collected over the previous 2 year.

The rupture risk of wide-narrow neck aneurysms is still controversial. Some studies found that high AR was positively correlated with aneurysm rupture (33) or narrow-necked aneurysms had higher risk of rupture (34). Other studies shown that wide-necked aneurysms were more likely to rupture (35). By comparing the results of most studies, we found that the mean AR value of ruptured aneurysms was mostly about 1.6, all in the range of wide-neck (33, 36). Yin et al. latest research found that non-linearity relationship of AR correlation to IA rupture, in the AR range of 1.08–1.99, the prevalence of IA rupture was lower for increment in AR, the prevalence of IA rupture increased in the AR range of 3.42–4.08, no association between AR and the prevalence of IA rupture in the AR range of 1.99–3.42 (37). In clinical practice, the AR of narrow-necked aneurysms with size <10 mm is rarely >3.42. The growth of aneurysm was accompanied by the change of a series morphology such as AR and hemodynamic (38). These studies showed that the rupture of aneurysm was the result of multiple factors. There may be some bias to analyze the risk of aneurysm rupture regardless of the size and shape. This may be one of the reasons why the current prediction model of aneurysm rupture is not ideal. In this study, the aneurysms were divided into wide-neck and narrow-neck, and the size of the aneurysms were set between 3 and 10 mm, which may help to reduce the bias of prediction model.

Previous research found that most studies used 10 mm as the cutoff point for size among other classes of aneurysms and that aneurysms larger than 10 mm rapture at a significantly higher (39, 40). Aneurysms smaller than 3 mm in diameter with less rupture risk, which was consistent with previous research (41). Therefore, we also chose the most common diameter of aneurysms ranging from 3 to 10 mm to reduce the deviation of the results.

Moreover, we found that RIAs had significantly higher IAP, WSS, WSSG, and lower NWSS than UIAs. When the aneurysms were divided into WNAs and NNAs, the RWNAs were found to have significantly higher IAP, WSS, and lower NWSS than the UWNAs. The RNNAs had significantly higher IAP and Streamwise WSSG, and lower NWSS. The results provide evidence that WNAs and NNAs have different hemodynamics, which could explain why the rupture ratios differ.

CFD is a popular research tool for studying hemodynamics; it utilizes 3D imaging data to perform numerical analysis and simulate intravascular blood flow structure to solve and analyze fluid flow problems. WSS is the tangential friction caused by blood flow on the arterial lumen. Several previous studies had examined WSS playing an important role in the natural process of IAs, however the results of WSS studies were inconsistent. Cebral et al. (42) demonstrated a positive correlation of WSS with aneurysm rupture and found that aneurysms with high WSS were more likely to rupture. Elsewhere, Castro et al. (43) built models of 26 anterior communicating artery aneurysms and discovered significant correlations between elevated maximum WSS with IAs rupture. Other studies, however, have found a link between low WSS and aneurysm rupture. Miura et al. (44) analysis 106 patient-specific middle cerebral artery (MCA) aneurysms found that only lower WSS was significantly associated with rupture status. According to an analysis of 119 aneurysm 3D angiographic images, Xiang et al. (13) discovered lower MWSS and WSS in RIAs compared to UIAs. Conflicting studies have linked high and low WSS to aneurysm rupture, resulting in the high- and low-stress theories. The high-stress theory believes that high WSS causes endothelial cell damage and initiates the degeneration of the vascular wall. Compelling evidence shows that when exposed to high WSS, vascular endothelial cells overexpress nitric oxide (NO), causing abnormally low arterial tone and apoptosis of smooth muscle cells (SMC) (45, 46). According to the low stress theory, low WSS causes inflammation of endothelial cells. Endothelial cells produce reactive oxygen species, which increase the adhesion molecules and cytokines on the surface of vascular walls, increase luminal permeability, and reduce the ability to withstand physiological hemodynamic forces (47, 48).

However, none of the previous studies classified aneurysms based on their neck wide. Numerous contradictory hemodynamic results have greatly reduced the predictive accuracy of hemodynamic parameters in aneurysm rupture. In this study, the WSS of RIA was significantly higher than UIA when there was no distinction between the wide neck and narrow neck. However, the WSS of RWNAs was significantly higher than UWNAs when distinguishing between the wide neck and narrow neck. Furthermore, WSS can be used as an independent predictor of WNAs using binary logistic regression. There was no significant difference in WSS between ruptured and unruptured groups in NNAs. The important finding was that WSS in UWNAs was higher than in RNNAs. Therefore, when analyzing the relationship between aneurysm rupture and hemodynamics, there will be a significant deviation in the results if the aneurysm is not classified by wide-neck and narrow-neck first.

The forced energy with which blood strikes the inner wall of the aneurysm sac is referred to as IAP. The local pressure rises at a flow impingement point as fluid kinetic energy is converted to static wall pressure (49). A high IAP area on the aneurysm wall may result in subsequent aneurysmal growth by a 6-month follow-up examination (50), increasing the risk of ruptured aneurysms. When the IAP reaches the critical value for aneurysm wall dilatation, the aneurysm will gradually expand, potentially leading to aneurysm wall thinning and rupture (51). Research evidence has demonstrated the effect of IAP on aneurysm rupture. Strategies, including aneurysm clipping or embolization, stent implantation were used to reduce IAP, which avoided aneurysm rupture intraoperatively and reduced aneurysm recurrence and rupture postoperatively. Yu and colleagues (52) investigated the hemodynamic changes in pre-and-post stenting of aneurysms and found that stent placement can reduce IAP and WSS at the neck region in side-wall aneurysms. In another investigation (53), the 4 different stents (LVIS, LVIS Jr, Leo, Barrel VRD) showed IAP decreased significantly after stent implantation. These findings suggest a correlation of IAP with aneurysm rupture. However, in the past, IAP was not considered a hemodynamic parameter for predicting aneurysm rupture.

In the present study, the IAP of ruptured aneurysms was found to be significantly higher than that of unruptured aneurysms in both the WNAs and NNAs groups. Also, binary logistic regression analysis revealed that IAP was an independent predictor of aneurysm rupture in both WNAs and NNAs. At the same time, the ROC curve analysis demonstrated that IAP is highly sensitive to predict aneurysm rapture in WNAs groups and NNAs group. This sensitivity is significantly higher than that used to predict aneurysm rupture risk based on factors, including age, hypertension, history of subarachnoid hemorrhage, aneurysm size, and aneurysm location (54). Meanwhile, there was no significant difference in IAP between RNNAs and UWNAs, indicating that if an aneurysm is not divided into WNAs and NNAs, the statistical results will be skewed. This paper show that the previous contradictory results of hemodynamics may be caused by the inaccuracy of the prediction model. If aneurysms are divided into wide-neck and narrow-neck aneurysms before hemodynamic analysis, it may greatly reduce the deviation of hemodynamic results, so as to more accurately guide the clinical strategies of pre-operation.

## Limitations

Although the study provided ideal models for removing the effects of patient-related factors, some limitations influenced the reliability of this study. The aneurysm size interval of 3–10 mm was selected; the interval was assumed to have only a minor effect, but it may also have caused deviations in the obtained results. Furthermore, the study was a retrospective analysis, and the CFD simulation used a patient-specific model. The blood flow in the hemodynamic simulation is assumed to be laminar and Newtonian, the walls are assumed to be rigid, and the inlet and outlet boundary conditions are non-specific to patients, which may result in deviations. Moreover, because our research facility is a large medical referral center and the majority of cases are referred by subordinate hospitals, selection bias is possible. In this view, the findings cannot be applied to other populations. Last but not least, the sample size of the present study was relatively small; therefore, additional multicenter studies with a larger sample size are needed to validate the study results.

## Conclusion

Wide-neck aneurysms and narrow-neck aneurysms have different hemodynamics, which could explain why they have different rupture ratios. This work has demonstrated that WSS and IAP are independent predictors of WNAs, but IAP is the only independent predictor of NNAs. These findings will assist neurosurgeons in developing more reasonable strategies for IAs before surgery.

## Data Availability Statement

The original contributions presented in the study are included in the article/supplementary material, further inquiries can be directed to the corresponding author.

## Ethics Statement

The studies involving human participants were reviewed and approved by Clinical Research Ethics Committee of Renmin Hospital. The patients/participants provided their written informed consent to participate in this study.

## Author Contributions

HW, QT, and ML contributed the conception, design, and drafted the manuscript. HW, KY, JW, PH, YG, WH, and WG contributed data acquisition and data analysis. HW, QT, YG, WH, and ML made the article preparation and editing and review. All authors contributed to the article and approved the submitted version.

## Funding

This research was funded by National Natural Science Foundation of China (Grant Nos: 81971870 and 82172173).

## Conflict of Interest

The authors declare that the research was conducted in the absence of any commercial or financial relationships that could be construed as a potential conflict of interest.

## Publisher's Note

All claims expressed in this article are solely those of the authors and do not necessarily represent those of their affiliated organizations, or those of the publisher, the editors and the reviewers. Any product that may be evaluated in this article, or claim that may be made by its manufacturer, is not guaranteed or endorsed by the publisher.
